# Health Bill brings NHS management back into government

**DOI:** 10.1136/bmj.s958

**Published:** 2026-05-15

**Authors:** Hugh Alderwick, Phoebe Dunn, Tim Gardner

**Affiliations:** Health Foundation, London, UK; Correspondence to: H Alderwick Hugh.Alderwick@health.org.uk

## Abstract

Concerns arise about political powers, patient voice, and data sharing

On 14 May, the UK government published a new Health Bill setting out the legislation ministers think is needed to deliver their plans for NHS reform in England.^1^ The bill follows government’s decision to scrap NHS England^2^—the national body responsible for day-to-day management of the NHS—and publication of its 10 year health plan in 2025.^3^ Awkwardly, the bill arrived just as the health secretary, Wes Streeting, resigned.

The government’s narrative is that the NHS must become more democratic to run effectively.^4^ But the legislation includes a range of changes to how the health service operates, spanning digital and data policy to the role of patients. Four issues require scrutiny from parliament.

First is the appropriate role for politicians in managing the NHS. The bill transfers a long list of powers from NHS England to the secretary of state—including on workforce planning, digital and data systems, oversight and regulation of local NHS bodies, and more. It also hands the secretary of state broad powers to direct local NHS planning decisions. Limited exceptions include individual clinical decisions or interventions on drugs and treatments that would be inconsistent with National Institute for Health and Care Excellence guidance.

Accountability for the health service will always rise upwards to politicians in a tax funded health system. But the bill hands them too much responsibility for its day-to-day management. For example, appointments of chairs and non-executive directors of local NHS bodies (who appoint their chief executives) have been managed at arm’s length of politicians since 2001.^5^ They will now fall to the secretary of state. The bill risks enabling excessive political interference that could cause harm—for instance, through political bias in appointing local leaders. Ministers must clearly articulate the expected benefits of these powers and stronger safeguards should be added—for instance, to keep some processes, such as appointments, at arm’s length. Ultimately, some split between policy and management should remain at the top of the NHS.^6^

Second is government’s logic for how the NHS will improve. The 10 year plan marked a return to market-style mechanisms that were a prominent feature of the last Labour government’s approach to managing the health service in the 2000s, with a sharper split between NHS commissioners and providers, greater freedoms for hospitals over finances, and measures encouraging competition between them (such as league tables).^7^ The bill legislates for some of these changes—for instance, revising NHS integrated care board (ICB) membership (to focus boards more squarely on the job of commissioning), and scrapping requirements for local NHS organisations to collectively manage their finances. Hierarchy, competition, and collaboration coexist in the NHS.^8^ But Labour’s reforms mean the dial is shifting away from collaboration as the organising principle for managing the health service.

The problem is that the big challenges facing health require a coordinated response. For example, the number of people living with multiple long term conditions is rising substantially.^9^ ICBs were originally designed to encourage cross-sector collaboration on these and other issues. Small changes to the bill could help—for instance, reversing the decision to remove mandatory local authority members from ICBs, given their responsibilities for public health and social care services that are essential for preventing disease and managing people’s health. In either case, evidence suggests that government’s plan to reshape ICBs as more traditional NHS commissioners is unlikely to have a major effect on how care is delivered.^10^ ^11^

## Risks for patient voice

Third is how to strengthen rather than diminish patient and public voice. The requirement for NHS foundation trusts to have councils of governors—a policy originally intended to boost accountability to communities—will be removed. And Healthwatch—the independent organisation that operates nationally and locally to gather patient and public views—will be abolished. The experience of Healthwatch has been varied and its impact hampered by budget cuts and a power imbalance with the NHS.^12^ ^13^ ^14^ But scrapping it entirely and bringing its functions “in house”^15^ means patients are losing an institutionally independent voice. The NHS has a mixed record of listening to patients,^16^ as repeated inquiries into patient safety failings show.^17^ ^18^ Government could choose to do more to listen to their views without reorganising anything. An independent body with sufficient infrastructure to gather local data and influence national decisions is needed.

Last is how the government’s plans for sharing patient data will work. The bill includes powers to establish a single patient record—bringing together existing information on people’s health and social care use into one place. The aim of joining up data to improve patient care is good. Patients and staff are frustrated with the current system, with clinicians navigating multiple and incomplete information systems.^19^ ^20^

But whether the plans can avoid the fate of past national NHS IT programmes^21^ is unclear. Detail on how the new record will work is thin. Questions include how it will combine existing fragmented systems and how the data will be accessed and governed, given concerns about NHS data sharing and privacy.^22^ Measures elsewhere in the bill to bring digital and data responsibilities into the Department of Health and Social Care may undermine the trust needed to deliver. Recent data suggest the public trusts the NHS more than the government with health data.^23^

The NHS is in the middle of yet another restructure. The bill brings the legislation needed to finish the job, but local NHS organisations are already in flux.^24^ The costs are clear: reorganisations eat up time and resources and can distract leaders from improving services.^25^ Potential benefits are harder to find—and the legacy of a more politicised NHS may prove poisonous.

## Notes

Provenance and peer review: Not commissioned; not externally peer reviewed.
